# Habitats as Surrogates of Taxonomic and Functional Fish Assemblages in Coral Reef Ecosystems: A Critical Analysis of Factors Driving Effectiveness

**DOI:** 10.1371/journal.pone.0040997

**Published:** 2012-07-16

**Authors:** Simon Van Wynsberge, Serge Andréfouët, Mélanie A. Hamel, Michel Kulbicki

**Affiliations:** 1 UR-CoRéUs, IRD (Institut de Recherche pour le Développement), Nouméa, New Caledonia; 2 UR-CoRéUs, IRD (Institut de Recherche pour le Développement), Laboratoire Arago, Banyuls/mer, France; Leibniz Center for Tropical Marine Ecology, Germany

## Abstract

Species check-lists are helpful to establish Marine Protected Areas (MPAs) and protect local richness, endemicity, rarity, and biodiversity in general. However, such exhaustive taxonomic lists (i.e., true surrogate of biodiversity) require extensive and expensive censuses, and the use of estimator surrogates (e.g., habitats) is an appealing alternative. In truth, surrogate effectiveness appears from the literature highly variable both in marine and terrestrial ecosystems, making it difficult to provide practical recommendations for managers. Here, we evaluate how the biodiversity reference data set and its inherent bias can influence effectiveness. Specifically, we defined habitats by geomorphology, rugosity, and benthic cover and architecture criteria, and mapped them with satellite images for a New-Caledonian site. Fish taxonomic and functional lists were elaborated from Underwater Visual Censuses, stratified according to geomorphology and exposure. We then tested if MPA networks designed to maximize habitat richness, diversity and rarity could also effectively maximize fish richness, diversity, and rarity. Effectiveness appeared highly sensitive to the fish census design itself, in relation to the type of habitat map used and the scale of analysis. Spatial distribution of habitats (estimator surrogate’s distribution), quantity and location of fish census stations (target surrogate’s sampling), and random processes in the MPA design all affected effectiveness to the point that one small change in the data set could lead to opposite conclusions. We suggest that previous conclusions on surrogacy effectiveness, either positive or negative, marine or terrestrial, should be considered with caution, except in instances where very dense data sets were used without pseudo-replication. Although this does not rule out the validity of using surrogates of species lists for conservation planning, the critical joint examination of both target and estimator surrogates is needed for every case study.

## Introduction

Among the existing conservation measures used to mitigate natural and anthropogenic impacts on marine ecosystems, the establishment of reserves are useful both to protect biodiversity and to sustain adjacent resources [1]. However, to be well designed and effective, reserves should be implemented using biological, social, and economic criteria, but this often requires a large amount of very specific data [2]. Considering biodiversity representativeness only, a comprehensive census of overall species richness, rarity, or endemism would be needed [3]. As ecosystem functioning depends more on functional traits than on species themselves [4], new conservation strategies promote the representation of functional groups, where important functional groups with little or no redundancy should warrant priority conservation effort. Unfortunately, costs of data acquisition, knowledge of species, and time limit taxonomic and functional inventories, which remain scarce. One possible approach to overcome this problem is to use surrogates [5]. The overall diversity of species and functional groups may remain unknown, but can be approximated by estimator surrogates variables that are more easily collected. These can be other taxa [6]–[7], environmental variables [8], and habitats [9]. In practice, surrogate-based conservation planning may require two steps. First, the effectiveness of various surrogates to represent the conservation target is evaluated using a reduced but representative data set. Second, if surrogacy is found sufficiently effective and sufficiently robust to sampling, the best surrogate can be used to search for new protected areas with confidence. At this stage, the surrogate is often spatially generalized and gridded at a given resolution by interpolation or modelling, if it is not already a gridded data set (remote sensing image for instance). Here, we focus on the first part of this two-stage process.

Surrogacy is only one of the tool available for conservation planning, which can be based on expert-opinion, customary rules, optimization of conservation costs for a given objectives and so forth [10]. Yet, surrogacy refers to date to a wide body of work [11]. In its simpler and more intuitive form, surrogacy is referring to the identification and use of surrogates data, instead of other data difficult to collect, for instance with statistical measurements of good-fit between the surrogate and the target (“pattern-based surrogate”). In its more achieved form, surrogacy is related to the use of “selection-based surrogates” to design a network of protected areas (a suite of locations of remarkable features) with a selection algorithm [12]. Our study is also related to the later domain.

One way to test if estimator surrogates are efficient for conservation planning consists in measuring to which extent a virtual reserve network established on surrogate data allow a good representation of the target data within the network [3], [6], [12], [13]. Using this approach (i.e. reserve selection algorithms) is interesting since algorithms maximize complementarity between selected sites. For example, prioritizing sites of high species richness only might result in a selection of sites containing similar subsets of species [14] but this would be avoided with a complementarity criteria. However, previous selection based studies have yielded highly variable effectiveness of surrogacy both between and within case studies. As a result, in their review paper, Favreau et al. (2006) [15] could not discern clear trends of surrogacy effectiveness due to high discrepancy of scales, taxa, and methods between studies. Rodrigues and Brooks (2007) [16] found more promising results, but surrogacy effectiveness appeared too variable to draw any decisive conclusion [11].

In coral reef ecosystems, conclusions remain largely variable. Beger et al. (2007) [6] found that corals, mollusks and fishes were not reliable surrogates for one another. In their review paper, Mellin et al. (2011) [14] found that surrogate effectiveness was the lowest for tropical coral reefs. Mumby et al. (2008) [17] found that selections of habitats designed to represent most of fish species richness were better surrogates for benthic species and overall biodiversity due to the wide distribution of fish across seascapes. In principle, habitats should be good surrogates of biodiversity since various theoretical (niche theory, island biogeography theory, etc) and empirical work show that areas of increasing richness of habitats house more species. Dalleau et al. (2010) [9] tested in Wallis Island if coral reef habitats were effective surrogates for fishes, invertebrates, corals, and algal species. They found variable effectiveness depending on habitat definitions, design algorithms used, and spatial scales, but concluded positively overall. Further tests in New Caledonia using fish data (M. Dalleau, S. A., M. K., unpublished data) and Maldives using a multi-taxa data set (S.A. and M.H., unpublished data) surprisingly led to different conclusions than in Wallis Island. Worse, the conclusions appeared extremely variables and contradictory between sites for similar configurations of taxonomic data sets and habitats. Facing such variability (and frustration), general patterns of surrogacy could hardly be drawn and preclude extrapolation of results from one site, one surrogate, and one spatial scale to others [12]. As a result, a significantly growing body of work could not yield better recommendations for conservation planning and management.

A better understanding of the drivers of the observed variability is required to make positive or negative recommendation for conservation planning. Looking back at a number of studies published so far, and looking at all coral reef studies, we realized that results have remained discussed from a broad statistical perspective, using the bulk of data, but never by dissecting the influence of particular stations, species, or spatial configurations. In fact, what was missing from the literature was a detailed step-by-step analysis of the reasons explaining the success or failure of a particular analysis, and a quantitative assessment of the influence of particular configurations.

To fill this gap, we considered here a New Caledonia site and evaluated the extent by which fish communities of specific locations could be represented by habitats around. A representative set of sampling sites was used to evaluate how various habitat descriptions at various scales could be used as surrogate of fish taxonomic and functional properties. Richness, diversity, and rarity of habitats were respectively evaluated as surrogate for fish richness, diversity and rarity. To explain why surrogacy is effective or not, results based on the entire data sets were compared with results based on reduced data set to assess the effect of each site. Then, effectiveness variability was critically assessed according to habitat definition and scale (estimator surrogates), the reference functional and taxonomic fish data set (target surrogate) and the conservation design (rarity, diversity and richness based algorithms).

## Methods

### Study Area

**Figure 1:**
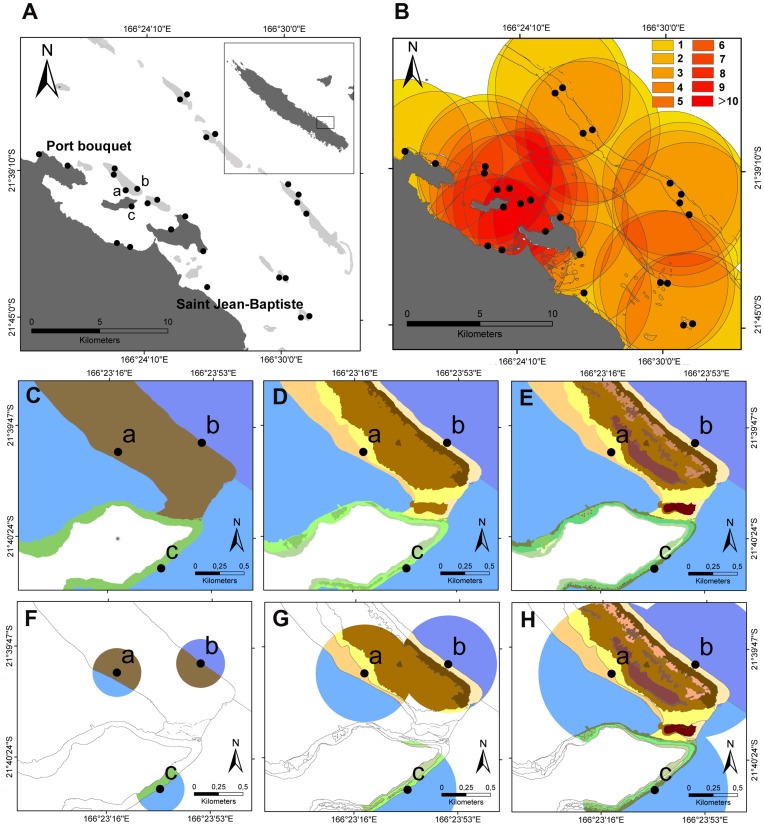
Characteristics of fish sampling stations. Panel **A** localizes sampling stations in Port-Bouquet bay in New-Caledonia. Black dots indicate fish census stations. Panel **B** indicates the number of overlaps between fish census’ 5000 m neighborhoods. For a specific area, the higher the number of overlaps (1 to more than 10), the higher is the bias of habitats’ rarity index. Panels **C–H** present habitat characterization around fish sampling stations 1, 2 and 3, based upon coarse geomorphology (C), a combination of coarse, medium, and fine geomorphology (D), and a combination of coarse, medium, fine geomorphology, rugosity, and benthic components (D). (F), (G) and (H) show habitats characterizing the three fish census stations when various grains are considered (respectively 250 m, 500 m, and 750 m).

New-Caledonia is a large western Pacific island, 1500 km east of Australia. Due to its coral reefs and lagoons, the Lagoons of New Caledonia became part of the UNESCO World Heritage list in 2008. The present study focused on Port-Bouquet Bay (Fig. 1a), where fishing activities are scarce and for subsistence only. This area is outside the UNESCO zones, yet its biodiversity is remarkable. It includes fringing reefs in protected bays and exposed to dominant tradewinds. Intermediate patch reefs are present in the deep open lagoon, which is bounded by barrier reef sections, both intertidal and subtidal. The entire domain covers 418 km^2^ of reefs and lagoons. Habitats include coral habitats, generally in good health but with some dead sections, several extensive algae-dominated reef flats, and multi-species seagrass beds. In the main bay, an existing MPA surrounds entirely a large high island.

### Fish Censuses and Functional Traits

Fish biodiversity data were collected in 2005, in the framework of the neo-Caledonian economic zone program (ZONECO). Presence/absence and abundance of 335 fish species were counted on 54 transects (50×10 m) distributed on 27 stations (Fig. 1a). Fish data were collected by underwater visual census using the method described in Labrosse et al. (2001) [18] and therefore did not require any ethical approval. In order to capture a significant fraction of the biodiversity of the area with a limited number of stations, sampling was stratified by reef geomorphology and exposure, which are known factors structuring fish communities [19]. Stations were selected *a priori* in the vicinity of coral habitat zones on three main reef types: fringing (9 stations), lagoon (10 stations) and barrier reefs (8 stations) (Table S1). This sampling design is the result of nearly 20 years of sampling effort in New-Caledonia and the Indo-Pacific and is optimized to describe fish diversity and abundance [20]. Similar stratification was also used to characterize fish community to support in 2008 the listing of New Caledonia reefs as UNESCO World Heritage areas [21]. More details on the underwater census procedure and sampling design are given in File S1.

Fish functional groups were defined afterwards by combining three life traits: trophic regime, maximum body size class (which determine the position of fishes in food webs), and mobility (which reflects fish home range) [22]. For trophic regime, we considered 4 qualitative classes (i.e. Plankton feeders; Herbivorous; Carnivorous; and Piscivorous). Mobility and size classes were semi-quantitative (six classes for body size and 4 classes for mobility). Combination of these classes led to define 59 functional groups for the study site and given the counted species.

### Habitat Mapping

#### In situ inventory and description of habitats

We described architectural and benthic characteristics from fieldwork at 36 seascapes selected from very high spatial resolution (2.4 meter) of Quickbird satellite images to capture most of habitat diversity in the study site [21]. A reefscape is defined here as a group of habitats, located in one broad geomorphological zone (e.g. a fringing reef, a patch reef, a barrier reef). A new habitat was recorded in the reefscape when coverage and/or architecture changed visually across several tens of meters. Thus, one reefscape site could yield several habitat descriptions (up to 9).

Architecture is related to the rugosity of the habitat (average variation of topography) but also to coral colonies growth forms and the height of seagrass and algal canopies. We used a Medium-Scale Approach (MSA) [23], to infer rugosity and coverage of benthic components for each sampled seascape and habitat.

#### Typology of habitats and mapping

We used 6 different ways to describe habitats, on the basis of geomorphology (coarse, medium, and detailed), topography, and benthic cover (coral and prevailing).

Three of them used geomorphological categories, reflecting distance to land, depth, and exposition to wind and swell. All these factors are potentially important to explain fish community structures [24]–[25]. First, a coarse characterization listed 8 broad classes of reefs (e.g. barrier reef; fringing reef, etc…). A medium characterization (16 classes) provided detailed geomorphological description (e.g. reef flat, internal slope, etc…) within each of the 8 coarse classes. A third geomorphological characterization (7 classes) referred to peculiar finer structures (e.g. vertical faults, spur and groves) which create many micro-habitats in terms of hydrodynamic, light and depth variations.

A fourth habitat characterization was based on rugosity only (i.e. local variation of topography) since rugosity is a primary structuring factor for fish communities [26], [27]. We ranked habitats into four classes of rugosity: low (variation of relief between 0 and 40 cm high above the floor), medium (between 40 and 100 cm), high (between 100 and 200 cm) and very high (>200 cm).

The next two habitat characterizations used benthic cover information. The fifth habitat characterization separated habitats with very low living coral coverage (<5%) from those with low (from 5 to 15%), medium (from 15 to 30%) and high living coral coverage (>30%). Then, the sixth characterization took into account groups of habitats defined from a Principal Component Analysis (PCA) of all benthic variables quantified by MSA.

Finally, 11 different typologies of habitats were defined by meaningfully combining these six characterizations (Table 1). Specifically, six simple typologies considered only one characterization at a time. The five last typologies successively combined coarse geomorphology with 1 to 5 of the other characterizations. The most detailed habitat definition was used to map habitat using a series of very high resolution Quickbird images at 2.4 m resolution. The user-oriented principles and methods from Andréfouët (2008) [28] were used to interpret the images. Then, for each typology, a map of Port-Bouquet Bay was achieved by degrading the information from the initial map (i.e. merging polygons with similar description) using the Geographic Information Software ESRI ArcMap 9.2 software. Figures 1c, 1d, and 1e illustrate three different maps, respectively on the basis of three different characterizations of habitats: from the simplest (coarse geomorphology only, Fig. 1c) to the most complete (Fig. 1e).

**Table 1:** Criteria used to characterize habitats in this study.

Habitatcharacterization	Geomorphology			Topography	Benthic cover	
	Coarse	Medium	detailed		Coral	Prevailing
1	•					
2		•				
3			•			
4				•		
5					•	
6						•
7	•	•				
8	•	•	•			
9	•	•	•	•		
10	•	•	•	•	•	
11	•	•	•	•	•	•

The first six characterizations consider only one criterion at time. The five last use various combinations of the six criteria.

### Testing Habitats as Surrogates for Fish Communities

#### Tested variables

To test whether habitats are good surrogates for fish communities, we calculated for each station fish richness, diversity, and rarity both for taxonomic groups (species) and functional traits. We also calculated habitat richness, diversity, and rarity for a given neighborhood. We tested 9 extents of neighborhood (circles of 30 m, 60 m, 100 m, 250 m, 500 m, 750 m, 1000 m, 3000 m, and 5000 m radius around each fish census station). Figures 1f, 1g, and 1h illustrate how habitats are taken into account for the surrogacy analysis around three fish sampled stations when different neighborhoods are used. With this spatial approach, we assumed that a fish assemblage sampled at a given station is dependent on the habitats around it. Indeed, most fish species we counted have home ranges larger than the transect length. For sedentary species, neighboring habitats might also influence nutrient flow, access to mobile prey, and in general provision of resources and therefore fish survival. We considered also very wide neighborhood (>1 km) in order to test the sensitivity of the surrogacy approach to various spatial scales, and to identify a possible optimal conservation unit size.

Richness was the number of species (or functional groups) and habitat richness was the number of mapped habitats around the station (or network of stations) for the neighborhood and the habitat typology considered.

For rarity, we created a rarity index for each entity (a species, a functional group, or a habitat) as a function of the number of sampled stations where the corresponding entity has been recorded (Table 2). Note that this rarity index is not independent of species richness and has not been previously reported in the literature. A rarity index is then calculated for each station (or network of stations) as the sum of rarity indices of all species/functional groups/or habitats present in the station or in the network (Table 2).

**Table 2:** Indices used to express rarity and diversity of habitats and fishes.

Index	Formula
Rarity index of fish i	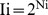
Fish rarity index of station/network k	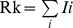
Rarity index of habitat j	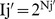
Habitat rarity index of station/network k	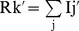
Fish diversity index of station/network k	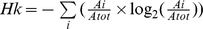
Habitat diversity index of station/network k	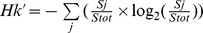

Hk is the Shannon-Weaver index of entity k.

Ni and Nj : Number of stations where the corresponding fish/habitat is absent.

Ai: Abundance of fish specie i.

Atot: Total abundance of all species.

Sj: Surface of habitat j.

Stot: Total surface of all habitats.

For diversity, the Shannon-Weaver index was calculated for each station (or network) by using fish abundances (*A_i_*) and habitat surfaces (*S_j_*) for the computations (Table 2).

Richness, rarity, and diversity of habitats were then respectively evaluated as surrogates of richness, rarity and diversity of fishes, both taxonomic and functional. The richness and rarity based algorithms are consistent with typical planning approach as they intend to protect as many entities (i.e. habitats, species or functions) and rare species as possible. The diversity based algorithm is more innovative. Its value is to protect as many entities as possible with an equivalent efforts: for example, protecting 10 species with similar effort might be far more interesting than protecting 1 species and barely protecting 9 others.

#### Surrogacy analysis

Iterative heuristic algorithms were performed (software R 2.13.0) to create MPA networks by selecting stations step by step according to their richness, diversity, and rarity of entities (habitats or fishes). Three algorithms were tested both on habitat data (surrogate scenario) and fish data (optimal scenario): i) A richness-complementarity algorithm selected stations to increase as fast as possible the number of entities included in the network. The first station selected was the richest. Stations iteratively added were those with the highest number of entities not already included in the network; ii) Then, a rarity algorithm selected step by step the richest stations in rare entities to add to the network; iii) Finally, a diversity-complementarity algorithm selected for each iteration the station that yielded the most diversified network (i.e., highest Shannon-Weaver index).

For each algorithm, the potential of habitats to be efficient surrogates of fish community was measured by comparing three curves:

The tested cumulative curve of richness, rarity or diversity of fishes included in the network when stations were selected by surrogate scenario (habitat data).
The optimal cumulative curve obtained when stations were selected by optimal scenario (fish data).
The average random curve obtained by 999 random selections of stations, framed by a 95% confidence interval.

We used an index similar to the Species Accumulation Index (SAI) previously described by Ferrier and Watson (1997) [8] to measure quantitatively the differences between these three curves:
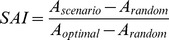
where *A_scenario_, A_random_* and *A_optimal_* were respectively areas under the tested curve, random curve, and optimal curve. A Species Accumulation Index close to 1 indicated that habitats were good surrogates of fish communities whereas Species Accumulation Index close to 0 indicated that the network of stations selected by habitat data did not reach its conservation goal faster than a random selection of stations, thus is a poor surrogate.

#### Factors driving variability

To identify the factors influencing effectiveness, we used the Sheirer-Ray-Hare extension of the Kruskall-Wallis’ test [29] to evaluate whether SAI results changed significantly between habitat characterizations, between neighborhood sizes, or both. We choose this test instead of analysis of variance because data were non-normal.

Robustness of surrogacy analysis to sampling was assessed by confronting (Wilcoxon Mann-Whitney paired tests) SAI results obtained with the entire data set (all stations) and results obtained when removing 1 to 5 randomly selected stations (99 runs). We then evaluated the proportion of simulations which provided significantly different results.

Finally, we summarize in Table 3 every scenario tested in this study. By combining all four factors (i.e. neighborhood size, habitat characterization, type of algorithm, and random removal of stations), 1782 scenarios were tested.

**Table 3:** Summary of all scenarios tested in this study.

Factors tested	Values tested
Conservation goal	Richness - Rarity - Diversity
Habitat characterization	1–2–3–4–5–6–7–8–9–10–11
Neighborhood size (m)	30–60–100–250–500–750–1000–3000–5000
Number of stations removed	0–1–2–3–4–5

One test corresponds to a particular combination of each factor and value.

## Results

### Fish Censuses and Habitat Mapping

335 species were counted among the 27 stations sampled, with a mean (SD) of 91 (20) species per station. Accumulation curves of fish species shows the level of biodiversity captured (Fig. S1). Spatial distribution was contrasted among species. Some genera (e.g., *Pomacentrus sp., Neopomacentrus sp.*) were highly abundant (more than 2000 individuals) at some stations but were absent at other stations. For six species, only one individual could be observed at only one station. Some other species (e.g. the butterflyfish *Chaetodon lunulatus* (Quoy & Gaimard (1825)) were widely distributed and observed at almost all stations. Similarly, some functional groups were highly redundant (i.e. 44% of groups were represented at more than 50% of stations) whereas others were found at only one station.

The most detailed habitat map encompassed 103 habitats classes, divided into 8 coarse geomorphological groups. Patch reefs, located 1 to 5 km offshore, were the most complex structures and could encompass 30 habitats overall. However, the richest fish sampling stations in terms of habitats (i.e. 40 habitats) were found on fringing reefs for a 5000 m neighborhood. At this scale, the station included fringing reef, barrier reef and patch reef habitats.

### Testing Habitats as Surrogates for Fish Communities

**Figure 2:**
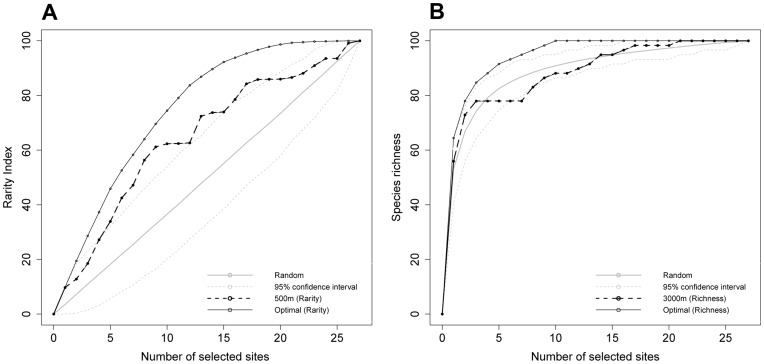
Accumulation curves of two contrasted examples of surrogacy effectiveness. A shows good surrogacy effectiveness obtained for functional rarity with a characterization of habitats based on medium geomorphology and considering a 500 m neighborhood size; **B** shows poor surrogacy effectiveness obtained for functional richness with a characterization of habitats based on topography and considering a 5000 m neighborhood size.

As expected, results of surrogacy effectiveness were contrasted. Figure 2 shows accumulation curves for two contrasted examples. The first one (Fig. 2a) was obtained for functional rarity, with a 500 m neighborhood size and using the medium geomorphological characterization of habitats. For this configuration, the tested cumulative curve of fish rarity index was above the average random curve and its 95% confidence interval, which implied good surrogacy effectiveness. The second example (Fig. 2b) is obtained for richness, with a 3000 m neighborhood size and using the rugosity based habitat characterization. Here, the tested cumulative curve of fish richness was below the average random curve and its 95% confidence interval, which implied poor surrogacy effectiveness.

**Figure 3:**
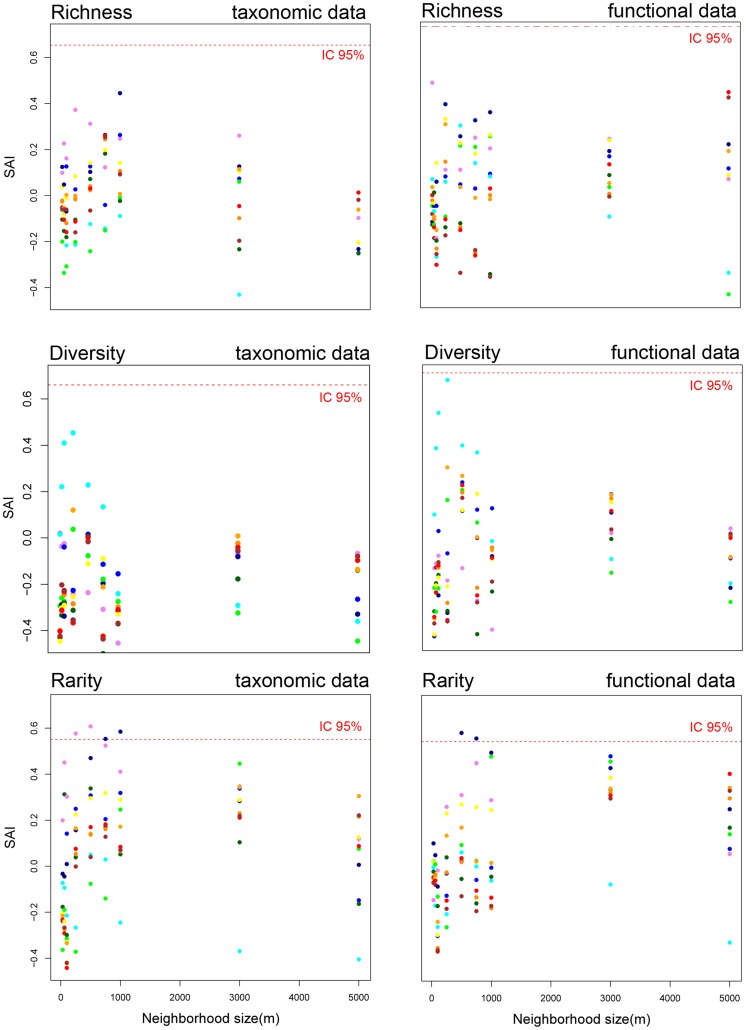
SAI results of all scenarios tested, for each neighborhood size and each characterization of habitats. SAI above 95% confidence interval are obtained for rarity only and mean that stations selected on habitats criteria are significantly better surrogates for fish communities than a random selection of stations.

Overall, the SAI results show that habitats were effective surrogate of fish communities in very few configurations (Fig. 3). Specifically, only the algorithm on the basis of rarity provided significant results (Fig. 3e and 3f). Those results were obtained for taxonomic data (Fig. 3e) when habitats were characterized by coarse and medium geomorphology, with neighborhoods of medium sizes (i.e. from 250 m to 500 m and from 750 m to 1000 m respectively). For functional data (Fig. 3f), best results were obtained when habitats were characterized by medium geomorphology, with neighborhood sizes of 500 to 750 m. Positive (although non-significant) results were also obtained for the diversity-based algorithm when combining a small neighborhood size (250 m) and a characterization of habitats based on rugosity. For the three algorithms, variability was high and was driven by the neighborhood size variation (Table 4). In addition, for the richness based algorithm, habitat characterization also influenced the spread of results (Table 4). Interaction between neighborhood size and habitat characterization was clearly visible on Fig. 3, but could not be tested statistically given the lack of replicates.

**Table 4:** Effect of habitat characterization and neighborhood extent on SAI results (Sheirer-Ray-Hare’s test).

Algorithm	Conservation target	Factor	H	p
Richness	Taxonomic groups	Habitat characterization	37.96	<0.001***
		Neighborhood size	23.58	0.003**
	Functional groups	Habitat characterization	23.56	0.005**
		Neighborhood size	17.08	0.030*
Rarity	Taxonomic groups	Habitat characterization	13.55	0.139
		Neighborhood size	26.52	<0.001***
	Functional groups	Habitat characterization	13.39	0.146
		Neighborhood size	32.87	<0.001***
Diversity	Taxonomic groups	Habitat characterization	13.55	0.139
		Neighborhood size	26.52	0.001***
	Functional groups	Habitat characterization	13.39	0.146
		Neighborhood size	32.87	<0.001***

Results are significantly affected by neighborhood size for all scenarios, and are affected by habitat characterization for richness scenarios only. Asterisks (*) indicate that results are significant with 95% (*), 99% (**), or 99,9% (***) confidence.

Importantly, removing stations from the initial data set affected SAI results, regardless of the variables tested (taxonomic and functional richness, rarity, or diversity). Removal of only one station could affect results significantly (Wilcoxon Mann-Whitney’s paired test, p<0.05) in 92 to 97% of the scenarios, all configurations included, for the three algorithms (Table 5). Conversely, the random removal of five stations significantly affected results only for 58 to 79% of simulations (Table 5). Most dramatic effects appeared with only one removal.

**Table 5:** Effect of removing stations on SAI results and comparison with all stations results (Wilcoxon’s test).

	Taxonomic data		Functional data	
Scenario	SAI Mean (SD)	% significantly different	SAI Mean (SD)	% significantly different
Richness				
All stations	−0.02 (0.19)		0.01 (0.21)	
1 station removed	−0.03 (0.20)	94%	−0.00 (0.20)	94%
2 stations removed	−0.02 (0.19)	81%	−0.01 (0.19)	72%
3 stations removed	−0.03 (0.19)	74%	−0.01 (0.18)	75%
4 stations removed	−0.02 (0.19)	77%	−0.01 (0.18)	69%
5 stations removed	−0.02 (0.19)	75%	−0.01 (0.17)	72%
Diversity	−0.00 (0.18)			
All stations	−0.19 (0.19)		−0.06 (0.17)	
1 station removed	−0.20 (0.18)	92%	−0.07 (0.22)	92%
2 stations removed	−0.20 (0.18)	84%	−0.07 (0.21)	80%
3 stations removed	−0.19 (0.17)	64%	−0.07 (0.21)	71%
4 stations removed	−0.20 (0.18)	66%	−0.08 (0.20)	81%
5 stations removed	−0.20 (0.17)	58%	−0.08 (0.20)	79%
Rarity				
All stations	0.05 (0.21)		0.04 (0.23)	
1 station removed	0.05 (0.26)	97%	0.03 (0.22)	95%
2 stations removed	0.06 (0.26)	81%	0.01 (0.22)	87%
3 stations removed	0.04 (0.26)	70%	0.02 (0.22)	82%
4 stations removed	0.05 (0.25)	80%	0.00 (0.21)	80%
5 stations removed	0.06 (0.25)	77%	0.01 (0.20)	70%

For all scenario tested (1 to 5 stations randomly removed from the initial dataset) we evaluated the proportion of SAI results significantly different from results obtained with the overall dataset. We also compared mean (SD) of SAI results when all neighborhood sizes and habitat characterizations are confounded.

## Discussion

The main goal of this study was to test if habitats could be effective surrogates of taxonomic and functional assemblages. We found generally weak effectiveness, except for some specific spatial scales and some habitat characterizations.

### Taxonomic Versus Functional Analysis

Ecosystem functioning is ruled more by functional assemblages than by taxonomic assemblages [4]. Changes in functional diversity are thus more likely to affect the stability, resistance and resilience of species assemblages than changes in taxonomic diversity [30], [31]. For these reasons, conservation planners should consider including functional criteria in their analysis to complement, or replace, taxonomic data [31]. Here, we found that habitats were generally weak surrogates for both taxonomic and functional fish data. For both, results were highly variable but we note that the best results were obtained for the same habitat typologies (i.e. coarse and medium geomorphology for rarity, rugosity for diversity) and for similar neighborhood distances (i.e. from 250 m to 1000 m). Finding similar general pattern for both types of data suggests that conservation plans built on taxonomic criteria could be transposed to functional criteria, and *vice versa.* However, even if taxonomic and functional analysis may share patterns, variability was high and taxonomic and functional fish data did not always provide the same range of Species Accumulation Index for a similar configuration of neighborhood sizes and habitat characterization.

### Factors Driving Variability of Surrogacy Effectiveness

Surrogacy analyses have been widely used and many studies report highly variable effectiveness results. Here we also found variable results according to neighborhood sizes and type of surrogates (i.e. habitat characterization) that match the variability reported both in marine and terrestrial ecosystems [6], [9], [13], [15], [17], [32]. However, none investigated systematically for a given data set the reasons that could explain the observed variability [12]. Ecological processes have been discussed to explain the results according to some feeding range, mobility, use of preferential habitats, and other ecological traits that could be related to the studied configurations, but no clear ecological and functional reasons could be sorted out. Specifically for the multi-taxa Wallis case study [9] and for the present results in fish communities, no known ecological processes can be identified to explain the patterns in Figure 3. Thus, here, we followed another path, by examining the influence of the randomly selected stations that have significantly changed the results when they were omitted from the analysis, for a given habitat configuration (habitat type and neighborhood size).

First, for richness-complementarity and diversity-complementarity scenarios, spatial distribution of habitats (specific to the study site) and spatial distribution of fish data (also specific to the study) drove habitat effectiveness as surrogate for fish communities. For neighborhoods large enough, several stations can yield a very high habitat richness, if, for instance, the station includes in this neighborhood different geomorphological zones. Typically, a station in an intermediate patch reef that happens to overlap a fringing reef for a given neighborhood size will have a very high habitat richness. If the distance between reef types is short, this will happen frequently, even for small neighborhood. Thus, between the many stations belonging to the same geomorphological structure, the algorithms will prioritize early in the iterations these stations close to different geomorphological zones. Station A was for example quickly selected by the diversity based algorithm for a neighborhood of 750 m, as this neighborhood covered both fringing and patch reef geomorphological structures (i.e. many habitats) (Fig. 2h). The algorithm then searched other type of complementary habitat configurations, likely around the barrier reef area, since barrier habitats were not yet included. Unfortunately, station A had very low fish diversity (H′ = 1.83 and H′ = 1.20 for taxonomic and functional diversity respectively), essentially dominated by one species: the coral demoiselle *Neopomacentrus nemurus* (Bleeker, 1857). For a habitat based algorithm, fringing reef fish species will thus be missing from the network since the following iteration favor missing complementary barrier habitat configuration, and the diagnostic is thus poor effectiveness. Considering a 750 neighborhood size, removal of station A thus changed the results from non-effective to effective (Wilcoxon Mann-Whitney’s paired test, p<0.05). This illustrates the significant influence of a single sampling station in testing surrogacy; and the importance of the reference data set sampling strategy (i.e. position of stations).

**Figure 4:**
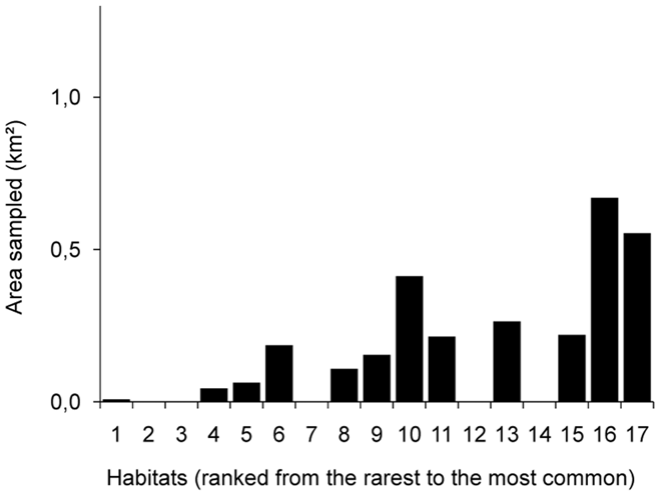
Discrepancies between habitat surface areas present on the area and habitat surface areas sampled. The figure displays a specific example, considering a 250 m neighborhood size and a characterization of habitats based on medium geomorphology. On the x-axis, habitats are ranked from the least (on the left) to the most (on the right) represented on the overall map. Some well represented habitats on the overall map (e.g. habitats “14” and “15”) appear under-sampled.

The rarity-based scenario first appeared interesting for management planning since best results of surrogacy were obtained for this scenario. However, the rarity-based algorithm was affected by sampling in 2 different ways. First, when stations are close enough spatially, their neighborhoods can overlap (e.g. Fig. 1h). If a rare habitat is present in that shared overlapping neighborhood, then this habitat is counted twice. The habitat rarity index is thus lower than what it should be. Increasing neighborhood size increases probability of overlapping and therefore increases the bias of the rarity index. A figure (Fig. 1b) shows oversampled areas, from this aspect, on the study site for a 5000 m neighborhood extent, which could possibly affect habitat’s rarity index. It is worth noting that oversampling is not here a replication issue. The choice of fish stations geographically close can be justified by different exposure to dominant wave and wind energy. Yet, this can affect the habitat rarity index as described above. Moreover, since these very close stations with similar habitat composition in their neighborhood could hold very different fish assemblages by design, this can explain the overall low surrogacy effectiveness we observed. Second, and for small neighborhoods, the surface areas of some habitats in the neighborhood of fish sampling stations were not representative of their overall presence on the map. Considering a 250 m neighborhood size and a habitat characterization based on medium geomorphology for instance (Fig. 4), the habitat characterization “intertidal reef flat” (numbered “15” on Fig. 4) is largely distributed on the overall map, but little represented in sampled sites’ neighborhoods. Thus, rarity is also very sensitive to the reference data set (e.g. fish) sampling design. Here, fish stations were located mostly on coral dominated areas (a frequent bias in fish survey, e.g. [6]) and rarity index of sandy, algal, or seagrass dominated habitats are overestimated compared to their actual coverage in the entire domain. To obtain less biased results of surrogacy analysis, stations should thus be sampled so that they best represent overall habitat surfaces and should be separated enough to avoid overlap between stations.

Thirdly, randomness affected selection of stations for richness and rarity based algorithm in the case of simple habitat characterization. When habitat characterizations were simple and neighborhoods were small, some stations could have exactly the same richness and rarity of habitats. In this case, stations were selected at random by the algorithm, which increased the variability of the surrogacy effectiveness depending on the fish community present on these stations. However, diversity based scenarios could not be affected by similar effects. Indeed, diversity accounts for the surface of the habitats a well, and it is unlikely that habitat surfaces around stations are perfectly equal for any neighborhood.

These effects of habitat spatial structure, sampling, and randomness were evidenced by investigating the strong shift in effectiveness when removing only one, or few, stations. Most striking is the influence that only one station could have, with conclusions on surrogacy effectiveness changing completely. By contrast, removing more stations does not necessarily affect results as much as only one station (Table 5). In fact, some stations increase effectiveness while others decrease effectiveness, thus removal of several stations annihilate their respective effect and has a lower effect on results overall. To our knowledge, this study is the first to report dependence of surrogacy analyses from so few stations in the marine environment, while identifying the reasons (spatial configuration of habitats, sampling) that explain the sensitivity. In a terrestrial environment, Freitag & Van Jaarsveld (1998) [33] assessed the effects of missing sites and missing taxa on reserve network design. They concluded that a 10–20% data deletion increased network variability (location of selected MPAs) and the number of marine protected areas required to represent all species sampled in the area.

The very low robustness of surrogacy analysis and the overall low surrogacy effectiveness we report here lead to cautious conclusions for conservation planning. As, in practice, MPA design is generally undertaken for one given scale and one given surrogate, sampling of the surrogate data set may therefore be extremely determinant on surrogacy effectiveness.

However, our sampling effort was moderate (only 27 stations). This likely amplified the importance of each station and limited robustness. Using a more extensive dataset should decrease the sensitivity to a particular set of stations. Nevertheless, the factors influencing effectiveness that we have identified in this study remain the same. Practitioners are now fully aware of some potential traps they should not forget, either when designing the sampling of the true surrogates, or when analyzing the results.

This study suggests that simulations need to be performed to assess if an optimal trade-off can be found to maximize robustness, in terms of estimator surrogate (habitat) description and neighborhood and target surrogate (fish) sampling efforts. Also, one can suggest having very few fish stations well separated spatially, sampled on habitats thematically and spatially apart. But limited stations likely imply an incomplete inventory, taxonomic or functional. By contrast, one can recommend the multiplication of sampling sites to limit the influence that only one site could have, but this may not be practical.

In the literature, for both pattern and selection based analysis, the range of sampling efforts implemented is wide. Most studies used larger data sets than ours [6], [7], [17], [34], [35], [36], [37] and should therefore remain reliable. For some other studies [9], [38], [39], [40], [41], the area sampled and the sampling effort is similar to ours and conclusions might need to be revisited. Some studies (e.g. [35], [40]) used sampling stations geographically very close (i.e. with similar habitat composition) and this might also affect surrogacy effectiveness measurements. When using only field data for diversity assessment, we believe that a high density of geographically distant sites is best to yield reliable results.

An alternative approach, widely documented in the literature, would be to divide the entire study area in virtual conservation units (i.e. grid cells), and to estimate species diversity of each cell by extrapolation of the reduced field data. With such an approach (which can be inspired by a variety of species distribution, habitat suitability, or niche models) surrogacy can be tested using grid cells (i.e., using the entire area) rather than being confined to sampling sites. Gridded methods, however, always generate unknown level of errors due to the extrapolation process, and may bias, positively or negatively, the interpretation of the true effectiveness of the surrogates. In fact, while many studies evaluated surrogacy effectiveness using extrapolated gridded approaches (e.g., [12], [13], [26], [41], [42], [43], [44], [45]), others directly used the sampling sites (e.g., [6], [8], [9], [39], [46]). Obviously, a site-based approach may not be entirely satisfactory for managers if they need a comprehensive spatial zoning plan. However, if the sampling sites were representative of the overall area, managers can be adequately guided. To our knowledge, the effectiveness of surrogates using site-based and gridded-based approaches has been seldom compared, and rather suggested higher effectiveness for gridded approaches [16]. This aspect warrants further investigations as it is an important unknown of the transferability of theoretical results to applied conservation planning.

### Using Habitats Maps for Conservation Planning?

Despite the intuitive interest to use habitat maps as surrogates of biodiversity census, we are still unable to tell whether habitats are effective surrogates of taxonomic and functional representation or not. In this study we showed contrasting results depending on the sampling effort achieved to build the reference data sets, against which effectiveness can be tested. We assume that similar conclusions could arise in other surrogacy studies, with different taxa and habitats, marine or terrestrial.

Consequences for conservation planning are two-fold. First, this clearly reinforces the idea that surrogacy results should not be extrapolated from one site to another, since various sets of sampling sites may provide highly different results [6], [13]. Secondly, we believe that surrogacy tests will be more reliable if a large amount of stations are sampled to build a reference. In such a case, each individual site would have a lower contribution to the overall diversity sampled, and more sites would be required to significantly change the results. If sampling effort is consistent enough, surrogacy analysis could be more influenced by the general pattern of habitat structure than the sampling and random effect. Our results suggest that the diversity algorithm is the least sensitive to the identified bias. It can be biased by a peculiar station located close to very different habitat configuration, but this configuration can certainly be easily avoided, if habitat maps (definitive or as pre-interpretation from a satellite image) are available beforehand. The best compromise to avoid bias seems to favor diversity-based algorithm, and achieve adequate sampling replication to avoid outliers.

Despite some encouraging results of habitats as surrogate for biodiversity [7], [9], conservation biology now faces a dilemma: on one hand, the high variability of surrogacy effectiveness among study areas implies to test surrogacy for each conservation plan [47], and remain reliable only for extensive sets of data, which involve both surrogate and target expansive censuses. On the other hand, surrogacy remains relevant only for broadly effective, affordable, and easily assessed surrogate. Considering time requirement for the task, we cannot expect managers to systematically test surrogacy effectiveness of different entities before starting conservation planning.

## Supporting Information

Figure S1 **A: Species accumulation curve obtained by iterative addition of random selection of stations (100 runs).** B: Gleason (1992) linear relationship between species richness and the logarithm of area sampled. In this model we considered a 2000 m^2^ area sampled by each station (2 transects of 1000 m^2^).
(TIF)

Table S1 **Number of fish sampling stations according to reef type and wind exposure.**
(DOC)

File S1 **Detailed description of the fish sampling design.**
(DOCX)
