# Supplementary figures and images for: Habitats as Surrogates of Taxonomic and Functional Fish Assemblages in Coral Reef Ecosystems: A Critical Analysis of Factors Driving Effectiveness
Source: PLoS One. 2012 Jul 16;7(7):e40997. doi: 10.1371/journal.pone.0040997 (PMC3397997; doi:10.1371/journal.pone.0040997)

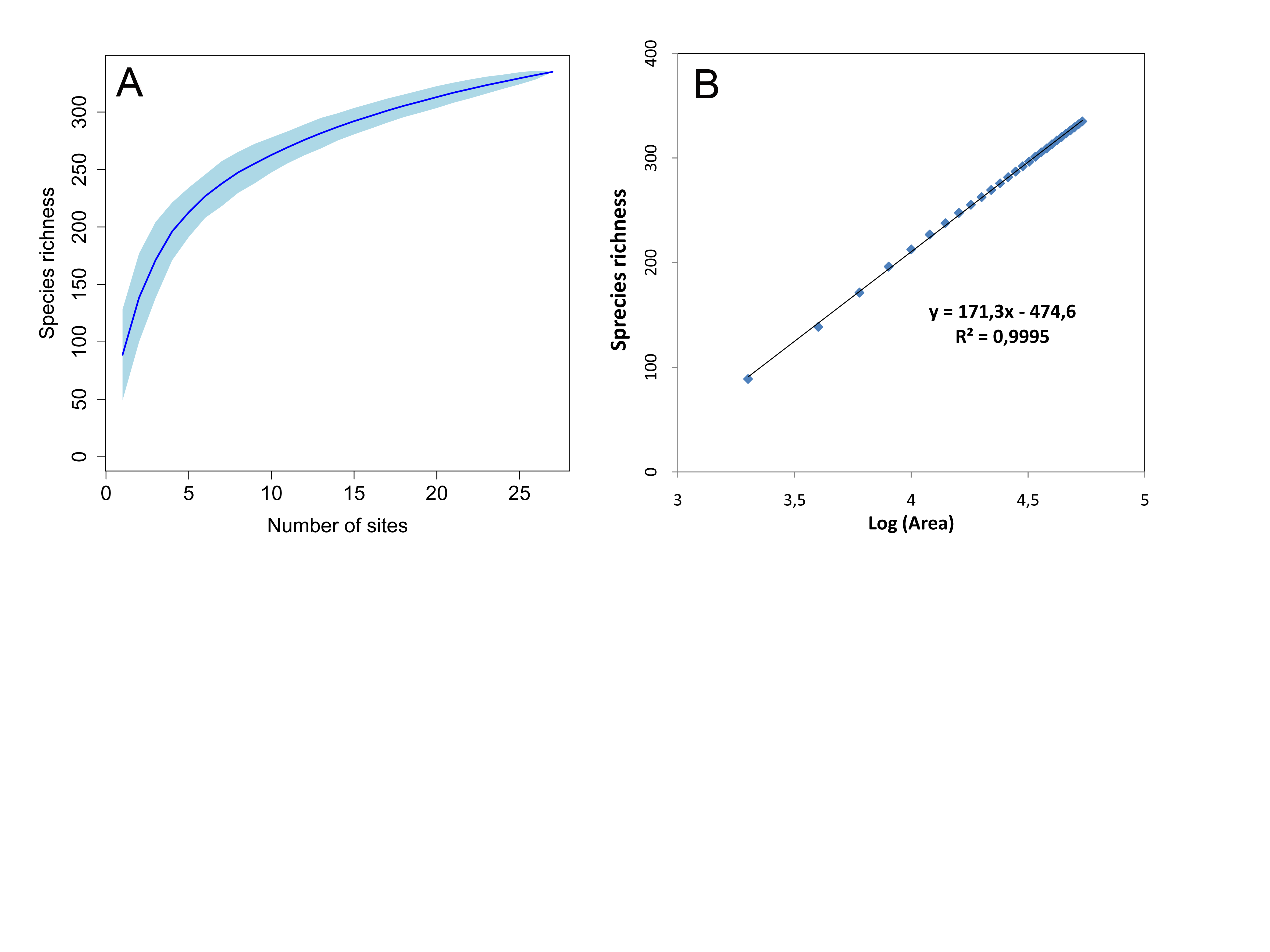

Supplement: Figure S1 — A: Species accumulation curve obtained by iterative addition of random selection of stations (100 runs). B: Gleason (1992) linear relationship between species richness and the logarithm of area sampled. In this model we considered a 2000 m2 area sampled by each station (2 transects of 1000 m2). (TIF) [file pone.0040997.s001.tif]
