# Supplementary material for: Habitats as Surrogates of Taxonomic and Functional Fish Assemblages in Coral Reef Ecosystems: A Critical Analysis of Factors Driving Effectiveness
Source: PLoS One. 2012 Jul 16;7(7):e40997. doi: 10.1371/journal.pone.0040997 (PMC3397997; doi:10.1371/journal.pone.0040997)
Supplement: Table S1 — Number of fish sampling stations according to reef type and wind exposure. (DOC) [file pone.0040997.s002.doc]

Table S1: Number of fish sampling stations according to reef type and wind exposure.

|  | Fringing | Intermediate | Barrier | Outer-Reef |
| --- | --- | --- | --- | --- |
| Leeward | 5 | 5 | 4 |  |
| Windward | 5 | 4 |  | 4 |
