# Supplementary material for: Habitats as Surrogates of Taxonomic and Functional Fish Assemblages in Coral Reef Ecosystems: A Critical Analysis of Factors Driving Effectiveness
Source: PLoS One. 2012 Jul 16;7(7):e40997. doi: 10.1371/journal.pone.0040997 (PMC3397997; doi:10.1371/journal.pone.0040997)
Supplement: File S1 — Detailed description of the fish sampling design. (DOCX) [file pone.0040997.s003.docx]

**Fish sampling design**

Reef fish assemblages were sampled on stations which were chosen to best represent the taxonomic and functional diversities. As for most of the Indo-Pacific, the reef fish assemblages in New Caledonia are organized along a strong gradient from the coast to the barrier reef (Kulbicki, 1988; Grimaud and Kulbicki, 1998). It is therefore important to stratify the sampling according to this gradient. One may consider 4 major reef types along this gradient, fringing, intermediate, barrier and outer reefs (Andréfouët et al. 2009) over most of New Caledonia’s main island. Trade winds (blowing from SE) are strong and frequent in New Caledonia, generating major differences in species distribution, leeward reefs being usually less diverse than windward reefs. Therefore the sampling of reef fish assemblages was also stratified according to this factor, which is important for intermediate and fringing reefs, but minor on outer reefs and barrier reefs. A total of 27 stations (Table S1) were sampled taking into account these two major factors.

On each station fish were recorded along a 50 m long transect line (2 transects per station). The method in use is described in detail by Labrosse et al. (2001) which is based on distance sampling theory (Buckland et al. 2004). The observers, one on each side of the transect line, record all fish (> 3 cm) they can detect. For each sighting the observers record the species name, the number of fish, their average size and the distance to the transect line. This method yields better results for large or active species, which usually have a large home range (Kulbicki, 1998; Kulbicki et al., 2010; Bozec et al. 2011), these species being usually the prime target of fisheries and therefore of protection (Pinca et al. 2011). This feature is important to take into account the patchy nature of reef fish distribution and allows to sample a wide corridor, usually up to 10 m on each side of the transect, and at times more, in particular when turbidity is very low (Kulbicki et al. 2010; Bozec et al. 2011). Therefore this sampling method covers a much wider area than most reef fish assemblage sampling methods which usually cover between 100 and 250m² per station, whereas here the area covered is between 500 and 1000m².

To evaluate the extent by which our sampling design represented the fish diversity of Port-Bouquet bay, we used species accumulation curves method with the Vegan 2.0-0 package in R 2.13.1 software (Oksanen et al. 2011). The curve is asymptotic (Fig. S1a) and indicates we sampled most of fish biodiversity. Gleason (1922) proposed a log-linear model in which the species richness is assumed to be a linear function of the logarithm of area sampled. This model fitted particularly well with our data (Fig. S1b) and indicated that a 50% increase of the number of stations would increase species richness of 37%. Sampling twice more stations than we did would be necessary to increase species richness of 66%. Moreover, such a value must be overestimated as Gleason’s model is built on the basis of a non-asymptotic equation.

**Literature Cited**

Andrefouet S, Cabioch G, Flamand B, Pelletier B (2009) A reappraisal of the diversity of geomorphological and genetic processes of New Caledonian coral reefs: a synthesis from optical remote sensing, coring and acoustic multibeam observations. Coral Reefs 28:691-707

Bozec YM, Kulbicki M, Laloë F, Mou-Tham G, Gascuel D (2011) Factors affecting the detection distances of reef fish: implications for visual counts. Marine Biology, 158:969–981

Buckland ST, Anderson DR, Laake JL, Burnham KP, Borchers DL, Thomas L (2004) Advanced Distance Sampling. Estimating abundance of biological populations. Oxford University press

Gleason, HA (1922) On the relation between species and area. Ecology 3, 158-162.

Grimaud J, Kulbicki M (1998) Influence de la distance à l’océan sur les peuplements ichtyologiques des récifs frangeants de Nouvelle-Calédonie – CRAS 321 : 923-931

Kulbicki M (1988) Patterns in the trophic structure of fish populations in the south-west lagoon of New Caledonia. Proceeding 6th International Coral Reef Symposium. Townsville 8-12 Aug. 1988, 2 : 89-94.

Kulbicki M (1998) How acquired behavior of commercial reef fish may influence results obtained from visual censuses. Journal of Experimental Marine Biology and Ecology 222 : 11-30

Kulbicki M, Cornuet N, Vigliola L, Wantiez L, MouTham G, Chabanet P (2010) Density estimates of coral reef fishes: transect length matters. Journal of Experimental Marine Biology and Ecology 387:15-23

Labrosse P, Kulbicki M, Ferraris J (2001) Underwater visual fish census surveys: Proper use and implementation. South Pacific Commission. Noumea. New Caledonia. 60pp.

Pinca S, Kronen M, Magron F, McArdle B, Vigliola L, Kulbicki M and Andréfouët S (2011), Relative importance of habitat and fishing in influencing reef fish communities across seventeen Pacific Island Countries and Territories. Fish and Fisheries. doi: 10.1111/j.1467-2979.2011.00425.x

Oksanen J, Blanchet FG, Kindt R, Legendre P, Minchin PR, O'Hara RB, Simpson RB, Solymos P, Stevens MHH and Wagner H (2011). Vegan 2.0-0, Community Ecology Package in R.
